# Association between novel inflammatory indices and osteoporosis among older adults: evidence from a large multicenter study in China

**DOI:** 10.3389/fmed.2026.1774083

**Published:** 2026-03-10

**Authors:** Ling Zeng, Hongyan Zhang, Meng Wang, Yu Zhang, Xiaoyu Zhang, Keli Li, Xiu Sun, Xi Chen

**Affiliations:** 1 Department of Traditional Chinese Medicine, Integrated Chinese and Western Medicine Diagnosis and Treatment Center, People’s Hospital of Xinjiang Uygur Autonomous Region, Urumqi, Xinjiang, China; 2 Heart, Lung and Vessels Center, Sichuan Provincial People’s Hospital, Chengdu, Sichuan, China; 3 Pain Diagnosis and Treatment Center, People’s Hospital of Xinjiang Uygur Autonomous Region, Urumqi, Xinjiang, China; 4 Department of Traditional Chinese Medicine, Xinjiang Medical University, Urumqi, Xinjiang, China; 5 Department of General Medicine, Huangshan City People’s Hospital, Huangshan, Anhui, China; 6 Department of Endocrinology, Chengdu First People’s Hospital, Chengdu, Sichuan, China

**Keywords:** inflammatory indices, multicentre study, older adults, osteoporosis, systemic inflammation

## Abstract

**Objectives:**

Chronic systemic inflammation is increasingly recognized as a key contributor to the development of osteoporosis. This investigation seeks to assess how effectively several inflammation-derived composite indices can diagnose osteoporosis in the elderly population.

**Methods:**

A multicenter cross-sectional study was conducted across four hospitals in China from January 2023 to May 2025, enrolling 3,625 participants aged ≥60 years. Associations between inflammatory markers and osteoporosis were examined with multivariable logistic regression, and potential nonlinear patterns were further investigated using restricted cubic spline (RCS) models. Diagnostic accuracy was assessed through receiver operating characteristic (ROC) curves and decision curve analysis (DCA).

**Results:**

Elevated values of several inflammation-related indices were associated with a higher likelihood of osteoporosis in multivariable analyses. RCS analysis demonstrated a nonlinear dose–response pattern (*p* for nonlinearity <0.001). These findings were consistent across stratified and sensitivity analyses. Among all indices evaluated, the aggregate index of systemic inflammation (AISI) exhibited the strongest association with osteoporosis (OR = 1.63; 95% CI: 1.49–1.78).

**Conclusion:**

This multicenter study demonstrates that elevated inflammatory indices are independently associated with osteoporosis, with AISI emerged as the superior marker, offering a novel, cost-effective tool for early identification of osteoporosis in clinical practice.

## 1 Introduction

Osteoporosis is a chronic systemic skeletal disorder, defined by decreased bone mineral density (BMD) and deterioration of bone microarchitecture, which together heighten bone fragility and fracture risk (1). With global population aging, the occurrence of osteoporosis has risen substantially in recent years (2). Globally, osteoporosis affects roughly 35.3% of older adult women and 12.5% of older adults men (3). Consequently, osteoporosis has emerged as a major global public health challenge, imposing a substantial burden on healthcare systems (4, 5). Therefore, early identification and timely intervention are crucial for reducing the disease burden and preventing adverse outcomes.

Osteoporosis has traditionally been attributed to age-related degeneration and hormonal deficiency. However, growing evidence highlights chronic systemic inflammation as a pivotal contributor to both the initiation and advancement of the disease (1, 6). Systemic inflammation disrupts bone remodeling by promoting osteoclast differentiation and activation while simultaneously impairing osteoblastic bone formation, processes that collectively drive accelerated mineral loss and deteriorate bone microarchitecture (7, 8). In light of inflammation’s central role…there is increasing interest in identifying practical and reliable biomarkers.

Composite inflammatory markers calculated from standard complete blood count parameters—including the systemic immune-inflammation index (SII), systemic inflammation response index (SIRI), monocyte-to-lymphocyte ratio (MLR), platelet-to-lymphocyte ratio (PLR), neutrophil-to-lymphocyte ratio (NLR), and the aggregate index of systemic inflammation (AISI)—provide a more comprehensive evaluation of overall systemic inflammatory load than single inflammatory indicators. Because they are calculated from low cost and widely available complete blood count tests, these indices are readily obtainable in clinical practice and are particularly suitable for large-scale population screening (9–13). Several studies have found that these indices are highly sensitive to inflammatory activity and are significant associations with the development and progression of multiple chronic conditions, such as cancer, metabolic syndrome, and cardiovascular diseases (9, 11, 12, 14). However, comparative evidence regarding the diagnostic performance of these inflammation-based composite indices remains limited, particularly in the older adults. To address this gap, we conducted a large, multicentre cross-sectional study to systematically assess and contrast the relationships between six such indices and osteoporosis risk in this population.

## 2 Materials and methods

### 2.1 Participant recruitment

**Figure 1:**
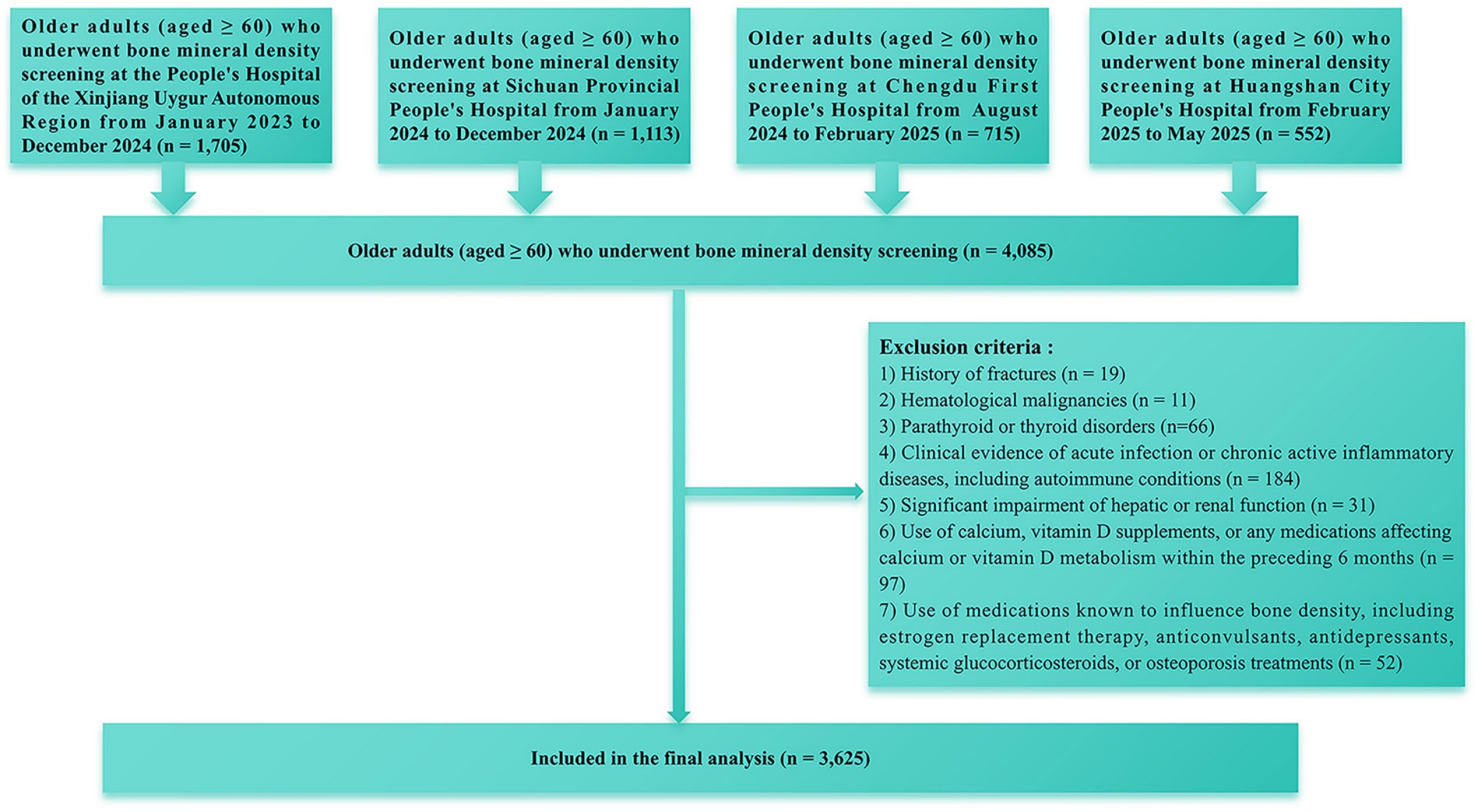
Flowchart for screening of study participants.

This multicentre cross-sectional investigation was carried out between January 2023 and May 2025. We initially recruited 4,085 participants aged ≥60 years from four medical centers in China. After applying the inclusion and exclusion criteria (Figure 1), 3,625 participants were ultimately included in the analysis.

### 2.2 Laboratory measurements

Demographic and clinical variables were obtained from the electronic medical record systems of the involved hospitals. The extracted information included age, sex, body mass index (BMI), blood pressure, lifestyle characteristics, coexisting diseases, and prior or current medication use. Peripheral venous blood was collected in the morning after an overnight fast, and an automated hematology analyzer was employed to quantify neutrophil, lymphocyte, monocyte, and platelet counts. Biochemical parameters—including alanine aminotransferase (ALT), aspartate aminotransferase (AST), high-density lipoprotein cholesterol (HDL-C), low-density lipoprotein cholesterol (LDL-C), total cholesterol (TC), triglycerides (TG), parathyroid hormone (PTH), fasting plasma glucose (FPG), and serum levels of potassium, calcium, phosphorus, and 25-hydroxyvitamin D—were assessed using a separate automated biochemical analyzer. Detailed descriptions of laboratory procedures, disease diagnostic standards, and medication categorization are presented in the Supplementary material.

### 2.3 Definition of osteoporosis

Osteoporosis was diagnosed using WHO criteria, characterized by a *T*-score ≤ −2.5 at the most severely affected site lumbar spine, femoral neck, or total hip—as outlined in the Supplementary material (15).

### 2.4 Inflammatory indices

The calculations of the inflammation indices are as follows (13):


$$\begin{array}{l} \text{AISI}=\text{Neutrophil count}\times \text{Platelet count}\times \\ \;\;\;\;\;\;\;\;\;\;\;\;\;\;\;\;(\text{Monocyte count}/\text{Lymphocyte count}); \end{array}$$

SIRI=Neutrophil count×(Monocyte count/Lymphocyte count);

SII=(Platelet count×Neutrophil count)/Lymphocyte count;

NLR=Neutrophil count/Lymphocyte count;

MLR=Monocyte count/Lymphocyte count;

PLR=Platelet count/Lymphocyte count.

### 2.5 Statistical analysis

Covariates were selected based on clinical relevance and prior literature review. Variance inflation factors (VIFs) were computed to screen for multicollinearity among covariates. Covariates with a VIF >5 were considered to exhibit multicollinearity and were excluded from the models (Supplementary Figure S1). The correlation analysis of the inflammatory indices is presented in Supplementary Figure S2. Relationships between inflammatory indices and osteoporosis were examined using multivariable logistic regression, with results reported as odds ratios (ORs) and 95% confidence intervals (CIs). To investigate potential nonlinear associations between these indices and osteoporosis risk, we fitted restricted cubic spline (RCS) models. We then quantified the ability of each inflammatory marker to discriminate osteoporosis using receiver operating characteristic (ROC) analyses and decision curve analysis (DCA). We additionally performed subgroup analyses and sensitivity analyses to examine the stability and reproducibility of the results. All statistical procedures were implemented in R software (version 4.5.1), with a two-tailed *p* < 0.05 regarded as statistically significant. Further methodological information is available in the Supplementary material.

## 3 Results

### 3.1 Baseline characteristics of participants

In total, 3,625 older adults were enrolled, of whom 1,010 had osteoporosis. Baseline characteristics are summarized in Table 1. Compared with individuals without osteoporosis, those with the disease were more frequently female and exhibited lower serum calcium levels (*p* < 0.001). Furthermore, all six composite inflammatory indices were markedly higher in participants with osteoporosis than in their non-osteoporotic counterparts.

**Table 1:** Baseline characteristics of the study participants.

Variables	Without osteoporosis	Osteoporosis	*p*-value
Participants, *N*	2,657	968	
Age (years)	69.00 (67.00, 72.00)	69.00 (67.00, 72.00)	0.556
Sex (%)			<0.001
Female	1717 (64.62)	445 (45.97)	
Male	940 (35.38)	523 (54.03)	
BMI (kg/m^2^)	25.34 (23.42, 27.41)	25.68 (23.53, 27.41)	0.289
SBP (mmHg)	143.00 (131.00, 156.00)	144.00 (132.00, 158.00)	0.111
DBP (mmHg)	92.00 (83.00, 101.00)	93.00 (84.00, 102.00)	0.056
Current smoking (%)			0.248
No	1,389 (52.28)	527 (54.44)	
Yes	1,268 (47.72)	441 (45.56)	
Current drinking (%)			0.953
No	1754 (66.01)	638 (65.91)	
Yes	903 (33.99)	330 (34.09)	
Inflammation index			
MLR	0.21 (0.17, 0.26)	0.24 (0.19, 0.29)	<0.001
PLR	121.95 (97.43, 154.08)	132.27 (106.47, 160.05)	<0.001
NLR	1.87 (1.46, 2.38)	2.16 (1.69, 2.75)	<0.001
SII	227.70 (153.11, 331.92)	295.23 (202.94, 466.34)	<0.001
SIRI	0.76 (0.58, 1.05)	1.07 (0.75, 1.51)	<0.001
AISI	183.72 (121.31, 277.53)	270.53 (189.43, 455.56)	<0.001
Biochemical index			
AST (mmol/L)	20.58 (18.11, 23.78)	20.34 (18.28, 23.32)	0.526
ALT (mmol/L)	29.29 (22.88, 37.81)	29.48 (23.00, 37.56)	0.911
HDL-C (mmol/L)	1.01 (0.87, 1.19)	1.01 (0.87, 1.20)	0.941
LDL-C (mmol/L)	2.55 (2.09, 2.98)	2.51 (2.07, 2.99)	0.581
TC (mmol/L)	4.35 ± 1.05	4.28 ± 0.98	0.072
TG (mmol/L)	1.59 (1.11, 2.35)	1.60 (1.03, 2.35)	0.133
PTH (pg/mL)	54.09 (40.90, 66.49)	54.20 (40.30, 69.60)	0.571
ALP (U/L)	71.00 (64.69, 78.90)	71.82 (65.45, 79.00)	0.086
Cr (μmol/L)	66.00 (55.70, 77.00)	66.85 (56.00, 77.50)	0.448
FPG (mmol/L)	5.06 ± 1.60	5.06 ± 1.61	0.985
Serum potassium (mmol/L)	3.85 (3.60, 4.12)	3.89 (3.64, 4.12)	0.122
Serum calcium (mmol/L)	2.26 ± 0.12	1.66 ± 0.12	<0.001
Serum phosphorus (mmol/L)	1.16 ± 0.17	1.14 ± 0.17	0.282
25-hydroxyvitamin D (nmol/L)	20.60 (11.34, 30.39)	21.24 (11.87, 31.85)	0.141
Medical history			
DM	773 (29.09)	294 (30.37)	0.455
Hypertension	1,400 (52.69)	539 (55.68)	0.110
CHD	293 (11.03)	105 (10.85)	0.878
Medications (%)			
Antihypertensive drug	1,159 (43.62)	449 (46.38)	0.138
Antiplatelet medication	969 (36.47)	323 (33.37)	0.084
Oral hypoglycemic drugs	318 (12.16)	102 (10.10)	0.082
Insulin	140 (5.27)	38 (3.93)	0.098
Statins	1,406 (52.92)	523 (54.03)	0.553

Data are presented as mean ± standard deviation, median (interquartile range), or percentages (%).

BMI, body mass index; SBP, systolic blood pressure; DBP, diastolic blood pressure; PTH, parathyroid hormone; ALP, alkaline phosphatase; ALT, alanine transaminase; AST, aspartate transaminase; Cr, creatinine; TC, total cholesterol; TG, triglyceride; HDL-C, high-density lipoprotein cholesterol; LDL-C, low-density lipoprotein cholesterol; AISI, aggregate index of systemic inflammation; SIRI, systemic inflammatory response index; SII, systemic immune inflammation index; NLR, neutrophil to lymphocyte ratio; PLR, platelet to lymphocyte ratio; MLR, monocyte to lymphocyte ratio; DM, diabetes mellitus; CHD, coronary heart disease.

### 3.2 Association between inflammatory indices and osteoporosis

**Figure 2:**
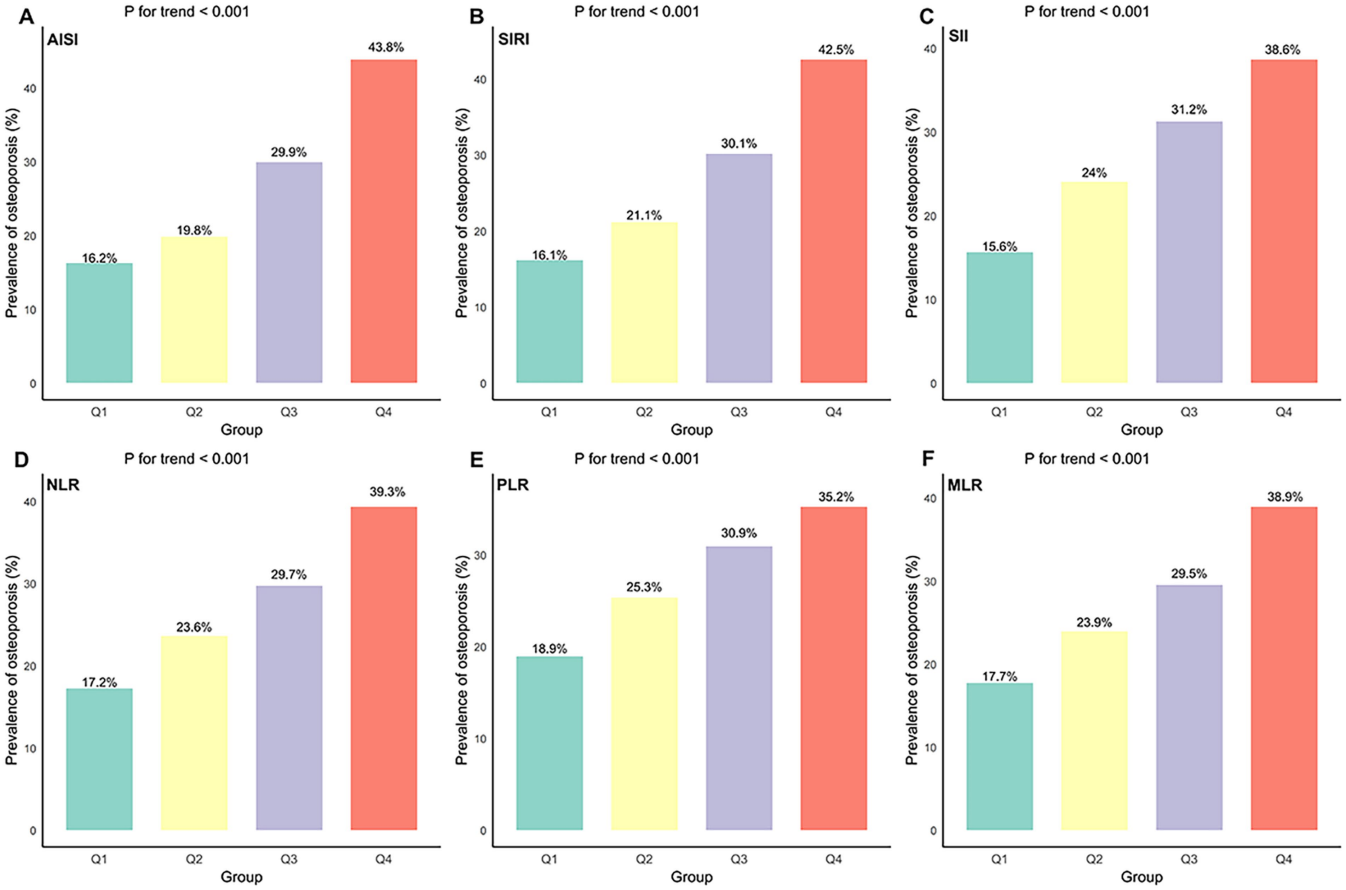
Prevalence of osteoporosis across quartiles of six inflammatory indices: **(A)** AISI; **(B)** SIRI; **(C)** SII; **(D)** NLR; **(E)** PLR; **(F)** MLR.

**Figure 3:**
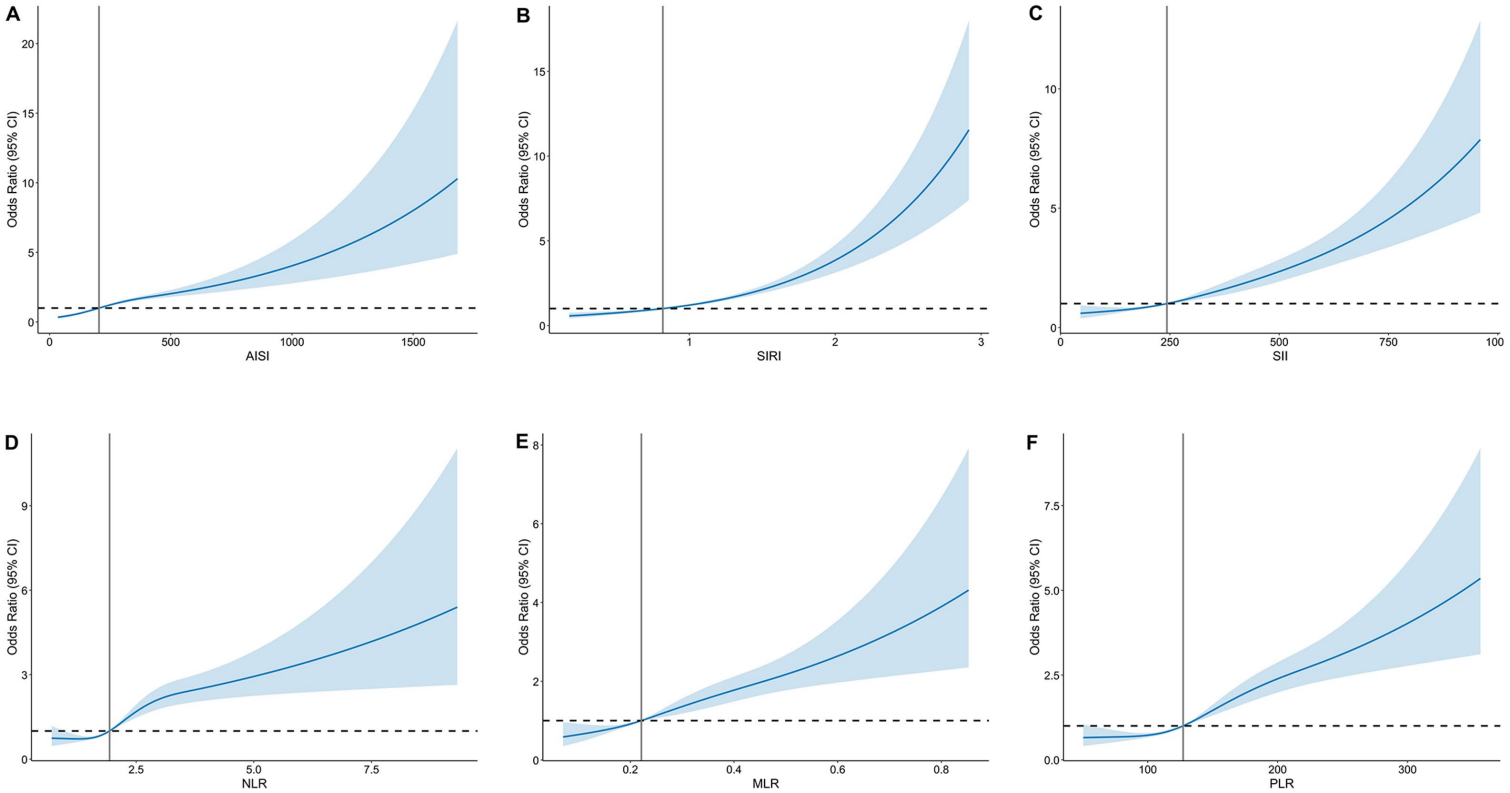
Dose–response association between inflammatory indices and risk of osteoporosis. **(A)** AISI; **(B)** SIRI; **(C)** SII; **(D)** NLR; **(E)** MLR; **(F)** PLR.

After stratifying participants into quartiles of each inflammatory index, we found that the prevalence of osteoporosis increased with higher quartiles (*p* for trend <0.001; Figure 2). In the fully adjusted multivariable logistic regression model, all six inflammatory indices were positively and significantly associated with osteoporosis (Table 2). When modeled as continuous exposures (per 1-SD increment), each index was associated with increased osteoporosis risk: AISI (OR: 1.63, 95% CI: 1.49–1.78), SIRI (OR: 1.54, 95% CI: 1.43–1.66), SII (OR: 1.49, 95% CI: 1.38–1.62), NLR (OR: 1.42, 95% CI: 1.33–1.52), MLR (OR: 1.36, 95% CI: 1.29–1.44), and PLR (OR: 1.23, 95% CI: 1.18–1.29). Consistent results were also observed after transforming continuous variables into categorical variables. Participants in the highest quartile (Q4) had substantially greater odds of osteoporosis than those in the lowest quartile (Q1), with OR of 3.83 (95% CI: 3.06–4.81) for AISI, 3.73 (95% CI: 2.98–4.67) for SIRI, 3.48 (95% CI, 2.76–4.38) for SII, 3.13 (95% CI, 2.51–3.91) for NLR, 2.90 (95% CI, 2.33–3.61) for MLR, and 2.40 (95% CI, 1.92–3.01) for PLR. In addition, RCS analyses revealed significant nonlinear associations between each inflammatory index and osteoporosis risk (*p* for nonlinear <0.001; Figure 3).

**Table 2:** Relationship between inflammatory indices and osteoporosis prevalence.

Exposure	Model 1	Model 2	Model 3	Model 4	Model 5
	OR (95% CI)	OR (95% CI)	OR (95% CI)	OR (95% CI)	OR (95% CI)
AISI (per SD increase)	1.65 (1.51–1.79)	1.64 (1.51–1.79)	1.63 (1.49–1.78)	1.63 (1.49–1.78)	1.63 (1.49–1.78)
AISI quartiles					
Q1 (<132.14)	Reference	Reference	Reference	Reference	Reference
Q2 (132.14–204.01)	1.30 (1.02–1.65)	1.30 (1.02–1.65)	1.29 (1.01–1.64)	1.29 (1.01–1.65)	1.28 (1.01–1.63)
Q3 (204.01–302.48)	2.29 (1.82–2.87)	2.28 (1.82–2.86)	2.26 (1.80–2.85)	2.24 (1.78–2.81)	2.23 (1.77–2.80)
Q4 (>302.48)	3.92 (3.15–4.89)	3.90 (3.13–4.87)	3.86 (3.08–4.84)	3.85 (3.07–4.83)	3.83 (3.06–4.81)
*p* for trend	<0.001	<0.001	<0.001	<0.001	<0.001
SIRI (per SD increase)	1.56 (1.45–1.67)	1.55 (1.44–1.67)	1.54 (1.43–1.66)	1.54 (1.43–1.66)	1.54 (1.43–1.66)
SIRI quartiles					
Q1 (<10.57)	Reference	Reference	Reference	Reference	Reference
Q2 (0.57–0.819)	1.40 (1.11–1.78)	1.40 (1.11–1.78)	1.40 (1.10–1.78)	1.39 (1.10–1.77)	1.40 (1.10–1.78)
Q3 (0.819–1.14)	2.32 (1.85–2.90)	2.30 (1.83–2.88)	2.27 (1.80–2.86)	2.25 (1.79–2.83)	2.25 (1.78–2.83)
Q4 (>1.14)	3.78 (3.03–4.72)	3.75 (3.01–4.68)	3.79 (3.02–4.74)	3.74 (2.99–4.68)	3.73 (2.98–4.67)
*p* for trend	<0.001	<0.001	<0.001	<0.001	<0.001
SII (per SD increase)	1.48 (1.37–1.60)	1.49 (1.38–1.61)	1.49 (1.38–1.62)	1.49 (1.38–1.61)	1.49 (1.38–1.62)
SII quartiles					
Q1 (<164.60)	Reference	Reference	Reference	Reference	Reference
Q2 (164.60–243.20)	1.71 (1.35–2.16)	1.72 (1.36–2.18)	1.71 (1.34–2.17)	1.71 (1.35–2.17)	1.71 (1.35–2.17)
Q3 (243.20–353.85)	2.50 (1.99–3.14)	2.53 (2.01–3.18)	2.50 (1.98–3.16)	2.50 (1.98–3.15)	2.51 (1.99–3.17)
Q4 (>353.85)	3.41 (2.72–4.26)	3.46 (2.76–4.34)	3.45 (2.74–4.35)	3.46 (2.75–4.35)	3.48 (2.76–4.38)
*p* for trend	<0.001	<0.001	<0.001	<0.001	<0.001
NLR (per SD increase)	1.42 (1.33–1.51)	1.42 (1.33–1.51)	1.42 (1.32–1.51)	1.42 (1.32–1.51)	1.42 (1.33–1.52)
NLR quartiles					
Q1 (<1.56)	Reference	Reference	Reference	Reference	Reference
Q2 (1.56–1.94)	1.49 (1.18–1.88)	1.49 (1.18–1.88)	1.49 (1.18–1.87)	1.49 (1.18–1.88)	1.49 (1.18–1.87)
Q3 (1.94–2.47)	2.03 (1.62–2.54)	2.03 (1.62–2.54)	2.00 (1.60–2.51)	2.01 (1.60–2.52)	2.01 (1.60–2.52)
Q4 (>2.47)	3.16 (2.54–3.93)	3.15 (2.53–3.92)	3.10 (2.48–3.87)	3.11 (2.49–3.89)	3.13 (2.51–3.91)
*p* for trend	<0.001	<0.001	<0.001	<0.001	<0.001
MLR (per SD increase)	1.37 (1.30–1.45)	1.37 (1.30–1.45)	1.36 (1.29–1.44)	1.36 (1.29–1.44)	1.36 (1.29–1.44)
MLR quartiles					
Q1 (<0.18)	Reference	Reference	Reference	Reference	Reference
Q2 (0.18–0.22)	1.44 (1.14–1.80)	1.44 (1.15–1.81)	1.43 (1.14–1.80)	1.43 (1.14–1.80)	1.43 (1.14–1.80)
Q3 (0.22–0.26)	1.90 (1.53–2.38)	1.91 (1.53–2.38)	1.90 (1.52–2.37)	1.89 (1.51–2.36)	1.89 (1.51–2.37)
Q4 (>0.26)	3.00 (2.42–3.72)	2.99 (2.41–3.71)	2.91 (2.34–3.62)	2.90 (2.33–3.61)	2.90 (2.33–3.61)
*p* for trend	<0.001	<0.001	<0.001	<0.001	<0.001
PLR (per SD increase)	1.22 (1.17–1.27)	1.22 (1.17–1.28)	1.23 (1.18–1.29)	1.23 (1.18–1.29)	1.23 (1.18–1.29)
PLR quartiles					
Q1 (<101.92)	Reference	Reference	Reference	Reference	Reference
Q2 (101.92–127.27)	1.47 (1.18–1.83)	1.48 (1.18–1.85)	1.47 (1.18–1.84)	1.48 (1.18–1.85)	1.51 (1.20–1.88)
Q3 (127.27–159.38)	1.99 (1.60–2.46)	2.02 (1.63–2.51)	2.05 (1.65–2.56)	2.05 (1.65–2.55)	2.07 (1.66–2.58)
Q4 (>159.38)	2.25 (1.82–2.79)	2.32 (1.87–2.89)	2.37 (1.89–2.98)	2.38 (1.90–2.98)	2.40 (1.92–3.01)
*p* for trend	<0.001	<0.001	<0.001	<0.001	<0.001

Model 1: unadjusted.

Model 2: age, sex, BMI, current smoking and current drinking, SBP, DBP.

Model 3: Model 2 plus adjustment for plus AST, ALT, HDL-C, LDL-C, TC, TG, PTH, ALP, Cr, FPG, serum potassium, serum calcium, serum phosphorus, 25-hydroxyvitamin D.

Model 4: Model 3 plus adjustment for DM, CHD, and hypertension.

Model 5: Model 4 plus adjustment for use of antihypertensive drug, antiplatelet medication, oral hypoglycemic drugs, insulin, statins.

SD, standard deviation; OR, odds ratio; CI, confidence interval. Other abbreviations are as defined in Table 1.

### 3.3 Inflammatory indices and their discriminative capacity for osteoporosis

**Figure 4:**
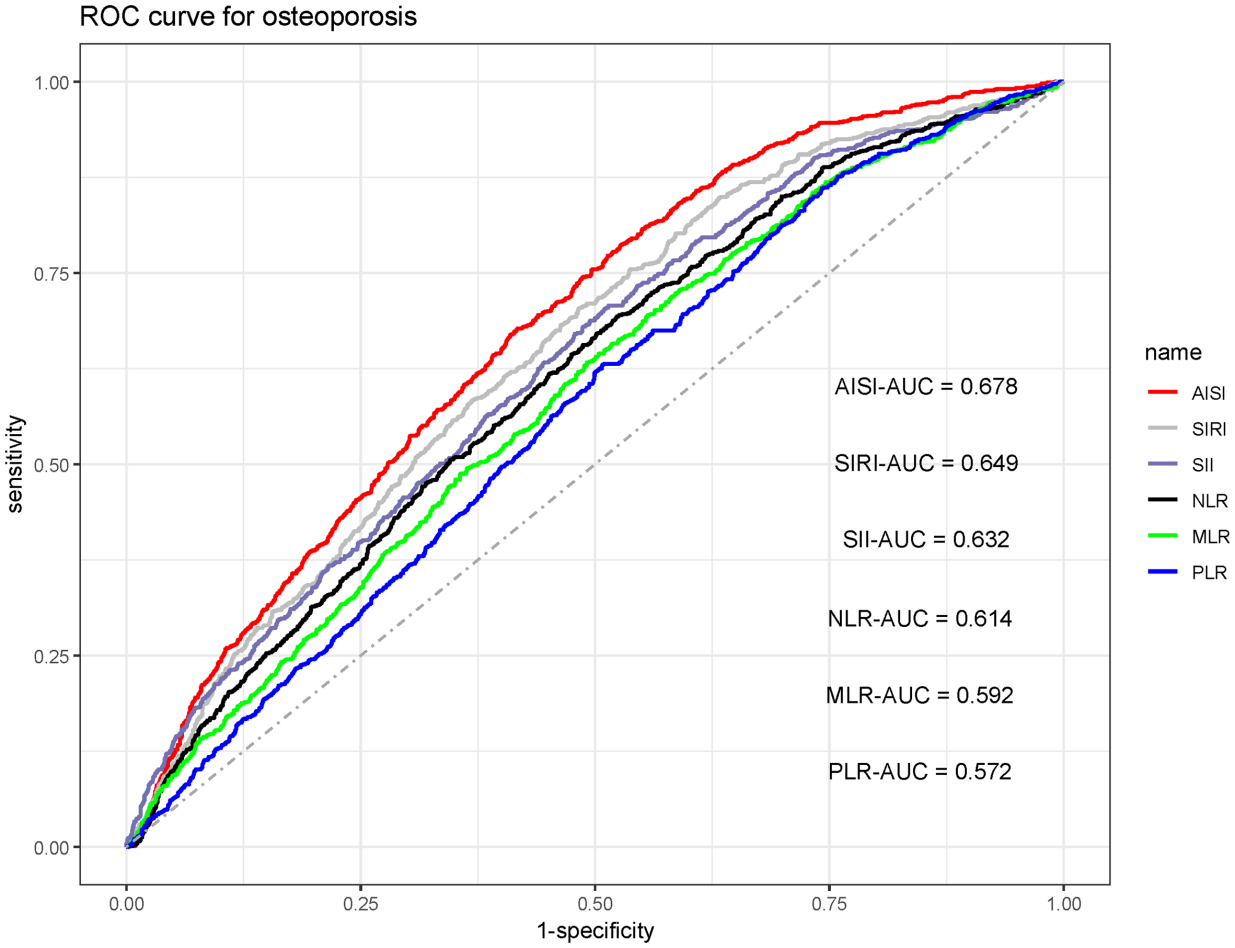
Comparative diagnostic utility of six inflammatory indices for osteoporosis: receiver operating characteristic (ROC) curves.

**Figure 5:**
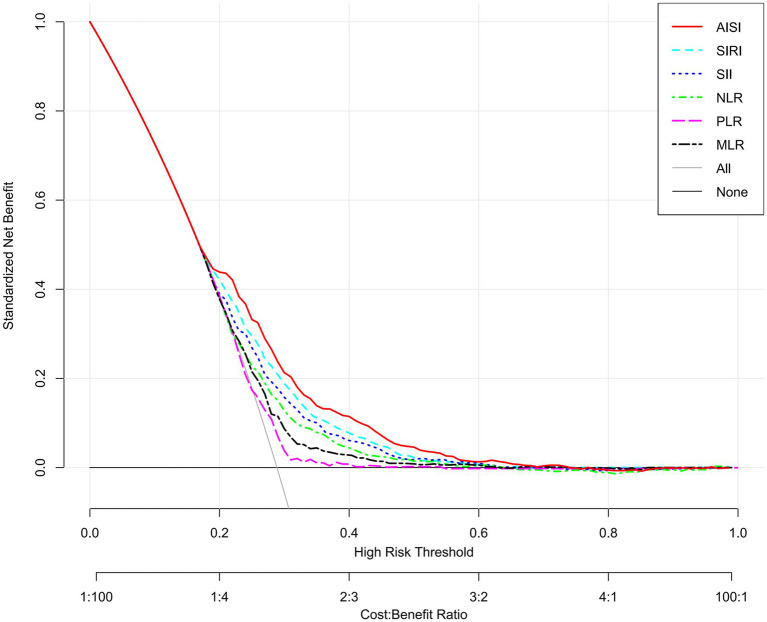
Decision curve analysis (DCA) for the diagnostic evaluation of osteoporosis using inflammatory indices.

The capacity of each inflammatory index to discriminate osteoporosis was assessed for each inflammatory index using the area under the receiver operating characteristic curve (AUC). As shown in Figure 4, AISI yielded the largest AUC (0.678), followed in descending order by SIRI (0.649), SII (0.632), NLR (0.614), MLR (0.592), and PLR (0.572). Subsequently, DCA was applied to compare the net clinical value of the six indicators. Consistent with the ROC-derived hierarchy, AISI provided the greatest net benefit for osteoporosis identifying osteoporosis, while PLR showed the lowest contribution (Figure 5). Additionally, we evaluated the incremental value of AISI beyond basic clinical variables. The base model including age, sex, and BMI yielded an AUC of 0.775 (95% CI: 0.758–0.792). Adding AISI significantly improved discrimination (AUC 0.804, 95% CI: 0.788–0.821; DeLong test *p* < 0.001, Supplementary Table S7).

### 3.4 Subgroup and sensitivity analysis

**Figure 6:**
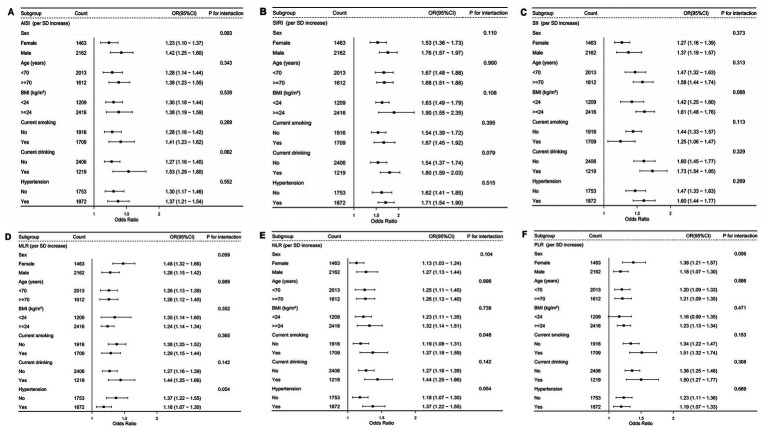
Association between inflammatory indices and osteoporosis in various subgroups. **(A)** AISI; **(B)** SIRI; **(C)** SII; **(D)** MLR; **(E)** NLR; **(F)** PLR.

To verify the stability of the results, we performed both subgroup and sensitivity analyses. Subgroup analyses by sex, age, current smoking, current drinking, and hypertension status showed consistent and statistically significant associations between inflammatory indices and osteoporosis risk across all strata, and no meaningful interactions between the indices and any subgroup variable were detected (all interaction *p* > 0.05; Figure 6). Additionally, a series of sensitivity analyses were performed. First, individuals with missing baseline covariates were removed to evaluate the influence of incomplete data (Supplementary Table S2). Second, participants with a BMI ≥30 kg/m^2^ were excluded to account for the potential confounding effect of obesity (Supplementary Table S3). Third, participants with diabetes mellitus (DM) were excluded to address the potential influence of chronic inflammation and abnormal bone metabolism associated with DM (Supplementary Table S4). Finally, participants with coronary heart disease (CHD) were excluded, given that CHD may be associated with elevated inflammation and could confound the observed associations (Supplementary Table S5). Stratified analyses by geographic region indicated no significant heterogeneity in the associations between systemic inflammatory indices and osteoporosis (all *p* for interaction >0.05; Supplementary Table S6). The results remained robust and consistent across all sensitivity analyses.

## 4 Discussion

In this large, multicenter cross-sectional study of 3,625 older adults, we evaluated the associations between six composite inflammatory indices and the risk of osteoporosis. Multivariable logistic regression indicated that elevations in each of the six inflammatory indices were independently linked to a higher likelihood of osteoporosis, and these relationships persisted in all subgroup and sensitivity analyses. Among the six markers, AISI exhibited the superior diagnostic performance, as corroborated by ROC and DCA analyses, highlighting its potential utility as a robust indicator for osteoporosis detection (Table 3).

**Table 3:** Summary of association strength and diagnostic performance of six inflammatory indices.

Inflammatory index	OR	*p*	AUC
AISI	1.63 (1.49–1.78)	<0.001	0.605
SIRI	1.54 (1.43–1.66)	<0.001	0.592
SII	1.49 (1.38–1.62)	<0.001	0.584
NLR	1.42 (1.33–1.52)	<0.001	0.575
MLR	1.36 (1.29–1.44)	<0.001	0.569
PLR	1.23 (1.18–1.29)	<0.001	0.558

Adjust: age, sex, BMI, current smoking and current drinking, SBP, DBP, AST, ALT, HDL-C, LDL-C, TC, TG, PTH, ALP, Cr, FPG, serum potassium, serum calcium, serum phosphorus, 25-hydroxyvitamin D, DM and hypertension, antihypertensive drug, antiplatelet medication, oral hypoglycemic drugs, insulin, statins.

SD, standard deviation; OR, odds ratio; CI, confidence interval. Other abbreviations are as defined in Table 1.

Osteoporosis is a chronic systemic disorder of the skeleton and a major contributor to fractures, disability, and increased mortality among older adults (16, 17). In addition, it is closely associated with a high incidence of cardiovascular and cerebrovascular diseases (18, 19). Given these multisystem consequences, greater emphasis on early detection and comprehensive preventive strategies is warranted.

Prior research has repeatedly demonstrated associations between inflammatory indices and osteoporosis. For instance, Ma et al. (5) reported that SIRI was significantly correlated with BMD and future fracture risk in elderly individuals with hypertension. Another investigation identified a positive link between increased SII levels and osteoporosis, proposing a threshold of 530.09 that yielded high sensitivity for identifying the disease (20). Analyses of studies retrieved from public databases further suggest that elevated SII and NLR are independently related to reduced BMD and a higher probability of osteoporosis among postmenopausal women (1). In addition, research in rheumatoid arthritis populations has shown that higher NLR, PLR, and MLR correspond to more rapid bone loss, a pattern similarly observed in postmenopausal women with diabetes (21, 22). Taken together, these studies highlight the pivotal role of inflammatory indices in both the pathogenesis and clinical identification of osteoporosis.

The present study demonstrates a robust positive association between systemic inflammatory markers and osteoporosis risk. Although the precise mechanisms remain incompletely characterized, multiple biological pathways are likely involved. Normal bone remodeling relies on a finely controlled equilibrium between osteoblast-driven bone formation and osteoclast-mediated bone resorption (23). Persistent inflammation gradually disturbs this equilibrium, promoting excessive bone resorption while suppressing bone formation, which in turn leads to progressive bone loss (24). Specifically, under stimulation by receptor activator of nuclear factor-κB ligand (RANKL) and macrophage colony-stimulating factor (M-CSF), monocytes may differentiate into osteoclasts, the principal precursor cells responsible for bone resorption—a key process in osteoporosis pathogenesis (25). Notably, intermediate monocytes show an increased propensity to differentiate into highly activated osteoclasts under inflammatory or stress conditions, thereby exacerbating bone resorption (26). Additionally, neutrophils and monocytes release various inflammatory mediators that substantially influence bone metabolism (27, 28). Among these, interleukin-6 (IL-6) regulates osteoclast differentiation by activating the RANK signaling pathway. Through this pathway, IL-6 enhances osteoclastogenesis and bone-resorptive capacity while simultaneously inhibiting osteoblast-mediated mineralization, tipping the balance toward net bone loss (29–31). Similarly, IL-1β strongly enhances RANKL-driven osteoclastogenesis by stimulating key transcriptional regulators such as NF-κB and activator protein-1, while also increasing C-C chemokine receptor type 7 expression, thereby facilitating osteoclast recruitment and activation (28, 32). Furthermore, TNF-α upregulates the CHIP E3 ubiquitin ligase, which induces degradation of osterix, a transcription factor critical for osteoblast differentiation, thereby impairing osteoblast differentiation, proliferation, and function, and ultimately inhibiting bone formation (32, 33). In addition, both IL-6 and TNF-α can induce the expression of endogenous antagonists of bone formation, including dickkopf-1 and sclerostin. These proteins inhibit the Wnt/β-catenin signaling pathway and consequently further limit osteoblast differentiation (34, 35). Notably, the relationship between inflammation and bone metabolism is likely bidirectional. While inflammation drives bone loss, osteoporosis itself may also promote a systemic inflammatory state. Research indicates that damage-associated molecular patterns (DAMPs), such as HMGB1 and S100 proteins released during bone matrix degradation, can activate Toll-like receptor (TLR) pathways (1, 36–38). This activation induces monocytes and macrophages to release pro-inflammatory cytokines like TNF-α and IL-6, potentially creating a vicious cycle (1, 36–38).

Our finding that AISI outperforms other indices in identifying osteoporosis may be attributed to its unique integration of the “coagulation-inflammation” loop. Unlike other markers, AISI encompasses all four key cellular effectors: neutrophils (inducing RANKL/IL-6 expression and osteoclast formation), platelets (promoting osteoclastogenesis), monocytes (driving osteoclastogenesis), and lymphocytes (immune regulation) (1, 39–41). This integrative mechanism provides a more comprehensive perspective on the systemic bone-resorptive burden. Although direct comparison with CRP was not possible in this dataset, existing literature suggests composite indices offer distinct advantages. While CRP is a well-established acute-phase reactant, its specificity for osteoporosis-related chronic “subclinical inflammation” is variable. Cellular immune dysregulation often precedes significant elevations in soluble markers (42). By capturing cellular effectors of inflammation and thrombosis, AISI may offer higher stability and superior sensitivity in detecting subclinical dysregulation (1, 39–41). Moreover, derived from routine complete blood counts, AISI represents a more accessible and cost-effective biomarker for population-level screening compared to high-sensitivity CRP assays.

Nonetheless, several limitations must be acknowledged. First, the cross-sectional design precludes causal inference. Although we observed associations between inflammatory indices and bone mass, reverse causation or shared underlying factors (e.g., frailty, immunosenescence) cannot be ruled out. Second, despite excluding participants with overt infections, unmeasured subclinical inflammation may still influence our results. Third, residual confounding from unmeasured variables (e.g., dietary calcium/vitamin D intake, physical activity) remains possible. Furthermore, due to the retrospective dataset, established inflammatory markers (CRP, IL-6) were unavailable, limiting direct comparison with AISI. Additionally, as our study sample was restricted to hospitalized older adults, the generalizability of our findings may be limited. Sixth, the lack of clinical outcome data (e.g., fracture events) precluded integration into risk tools like FRAX. Finally, specific geriatric metrics (e.g., frailty scores) could not be retrospectively assessed. Future prospective studies with comprehensive clinical outcomes, longitudinal follow-up, and mechanistic evaluations are warranted to validate our findings and explore the translational potential of inflammatory indices in osteoporosis assessment.

## 5 Conclusion

Our results demonstrate that the aggregate inflammatory score index (AISI) exhibited superior diagnostic performance in identifying osteoporosis than other inflammatory indices markers, highlighting its promise as a biomarker for clinical identifying osteoporosis. However, additional studies are required to validate these results.

## Data Availability

The raw data supporting the conclusions of this article will be made available by the authors, without undue reservation.

## Supplementary material

SUPPLEMENTARY FIGURE S1 Collinearity diagnosis.

SUPPLEMENTARY FIGURE S2 Spearman correlation matrix of six inflammatory indices.
