# Supplementary figures and images for: Association between novel inflammatory indices and osteoporosis among older adults: evidence from a large multicenter study in China
Source: Front Med (Lausanne). 2026 Mar 10;13:1774083. doi: 10.3389/fmed.2026.1774083 (PMC13008944; doi:10.3389/fmed.2026.1774083)

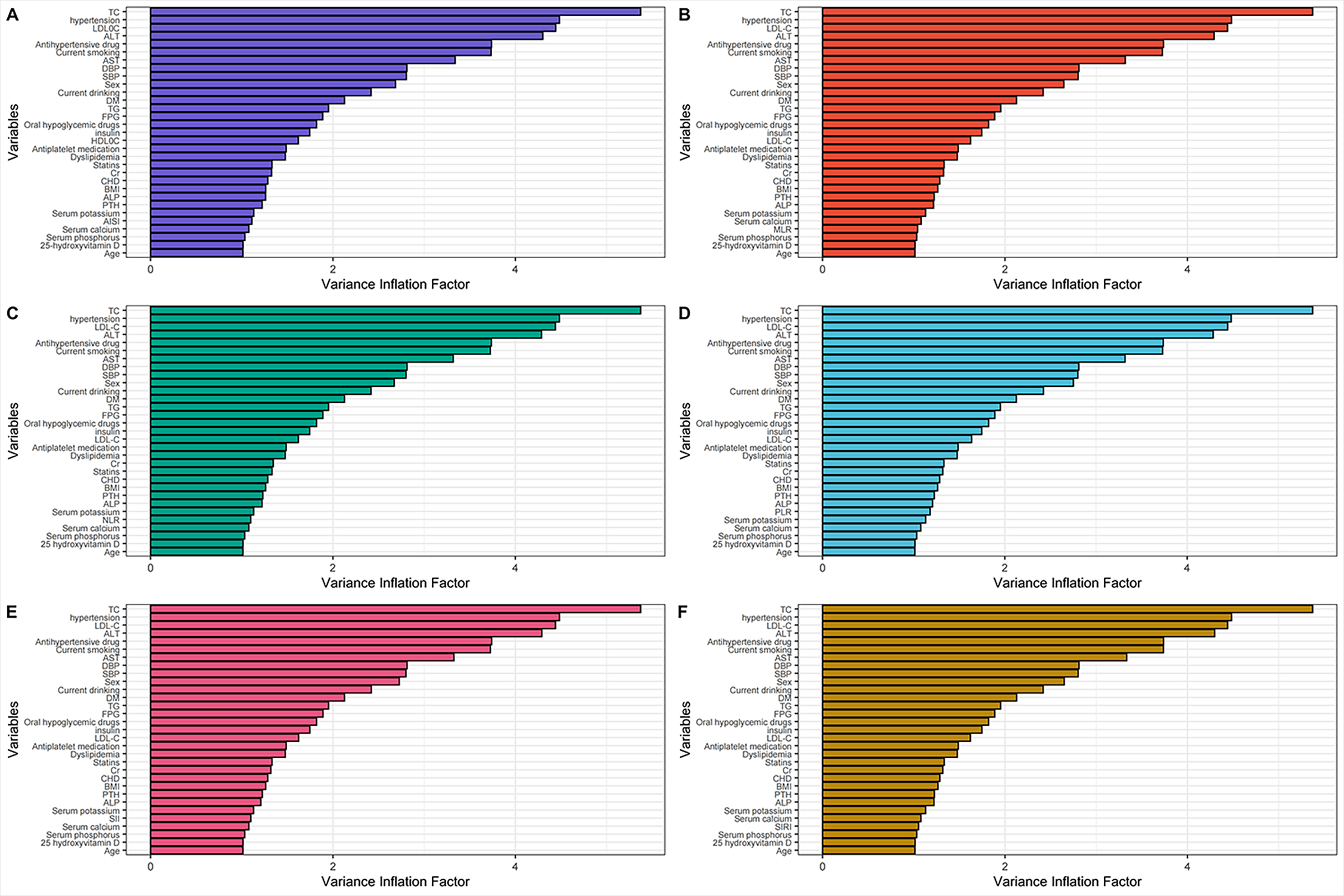

Supplement: SUPPLEMENTARY FIGURE S1 — Collinearity diagnosis. [file Image_1.TIF]

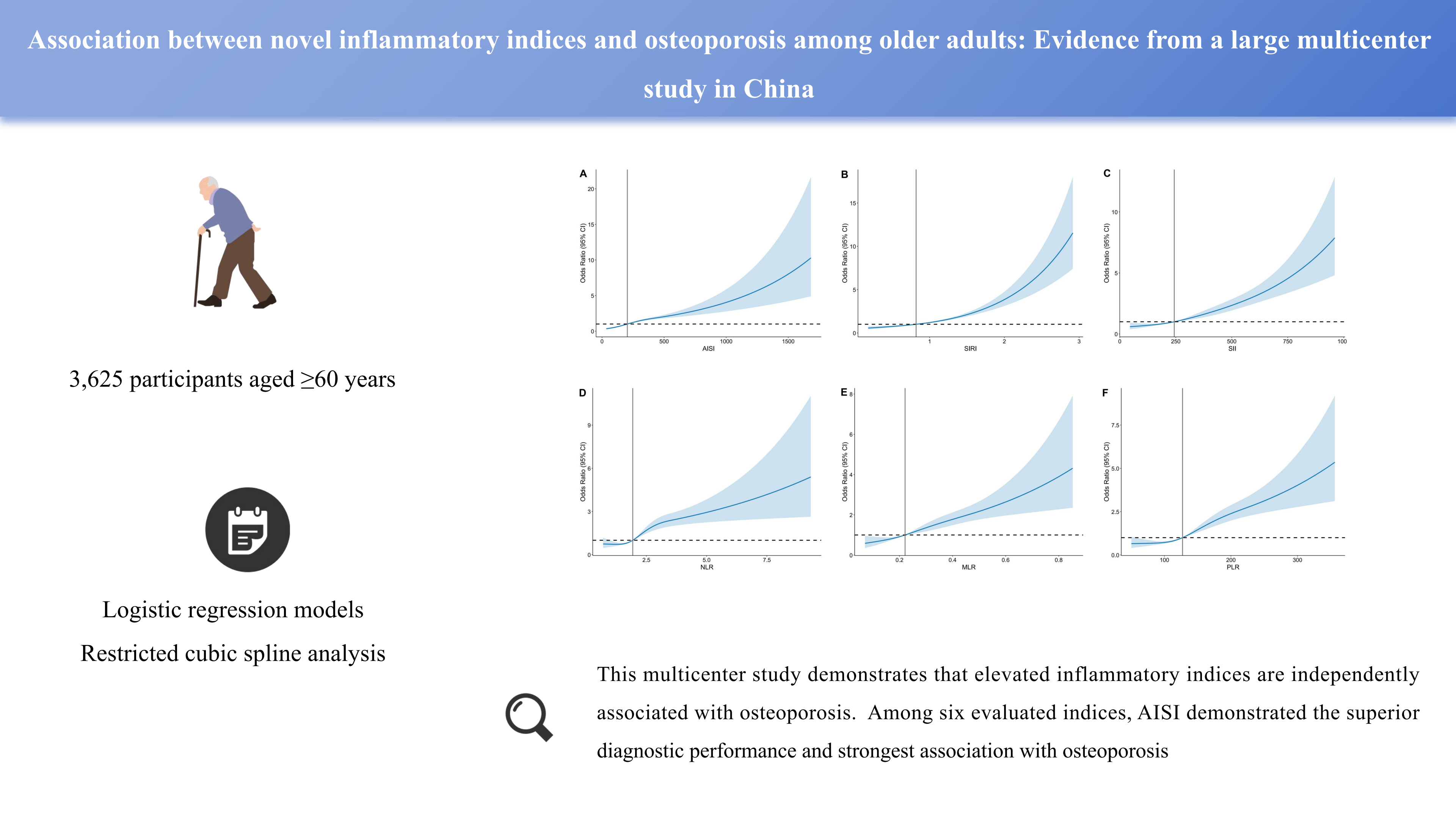

Supplement: SUPPLEMENTARY FIGURE S2 — Spearman correlation matrix of six inflammatory indices. [file Image_2.TIF]
