# Supplementary material for: Association between novel inflammatory indices and osteoporosis among older adults: evidence from a large multicenter study in China
Source: Front Med (Lausanne). 2026 Mar 10;13:1774083. doi: 10.3389/fmed.2026.1774083 (PMC13008944; doi:10.3389/fmed.2026.1774083)
Supplement: Supplementary file 3 [file Data_Sheet_1.docx]

Supplementary Material

**1 Supplemental material and methods**

**Baseline examination**

Anthropometric data (weight and height) and blood pressure were collected by trained nurses. Using a standardized protocol, body weight (kg) was measured to the nearest 0.1 kg, whereas height (cm) was measured to the nearest 0.1 cm in bare feet. Body mass index (BMI) was calculated according to the formula: BMI = weight in kg/(height in m)2. Current smokers were defined as having smoked 100 cigarettes in their lifetime and currently smoking. Current drinking was evaluated with questions regarding the types of alcoholic beverages, the frequency of alcohol consumption per week, and the usual amount consumed per occasion. Subjects who reported alcohol consumption >140 g/week for men and >70 g/week for women were deemed to have excessive alcohol consumption^1^ .Blood pressure was measured using a mercury sphygmomanometer after the patient had rested quietly for at least 10 minutes, and the average of multiple measurements was taken as the systolic and diastolic blood pressure values.

**Bone Mineral Density Assessment**

Bone mineral density (BMD) was measured at the lumbar spine (L1–L4), femoral neck, and total hip using dual-energy X-ray absorptiometry (DXA). The participating centers utilized three types of DXA scanners: the Hologic Horizon Wi (Hologic Inc., MA, USA), the GE iDXA (GE Healthcare, USA), and the GE DPX-NT (GE Healthcare, USA). The DXA machine was calibrated daily with a phantom. Lumbar spine (L1–L4) and femoral regions (total femur; femur Neck; Ward's triangle) scans of each subject were performed and analyzed upon test completion, with each scan taking approximately 5 min. The short-term in vivo coefficient of variation for the DXA machine was 1.8% for the lumbar spine and 1.2% for the femoral regions. The lumbar and femoral regions BMD were computed automatically by the DXA scanner. The precision error (coefficient of variation) was consistently low (<2.0%), ensuring measurement reliability within each center. Vertebral levels with structural artifacts (e.g., compression fractures, severe osteophytes, or metal implants) were excluded from the analysis to ensure the validity of the T-scores.

**Definitions**

Diabetes mellitus was defined as fasting serum glucose ≥7.0 mmol/L, the 2-h serum glucose of the oral glucose tolerance test ≥11.1 mmol/L, or the current use of hypoglycaemic medication or insulin. Coronary heart disease (CHD) was defined as myocardial infarction, unstable angina, and coronary revascularization. Hypertension was defined as a systolic blood pressure of 130 mm Hg or higher, a diastolic blood pressure of 80 mm Hg or higher, or treatment with antihypertensive agents.

**Details of the statistical analyses**

Variables of baseline characteristics are shown as n (%) if categorical, mean (SD) if normally distributed. In order to compare features between the two groups, Chi-square test was used for categorical variables, and one-way analysis of variance, or the Kruskal-Wallis test was used for continuous variables with normal and skew distributions. To minimize bias caused by missing values, we employed Multiple Imputation by Chained Equations (MICE) rather than simple interpolation. First, variables with missing rates greater than 20% were excluded from the analysis. Subsequently, we utilized the mice package in R software to generate 5 imputed datasets for the remaining variables. For continuous variables, predictive mean matching (PMM) was used to ensure the imputed values fell within the observed range of the original data. Prior to any regression analysis, we tested multicollinearity among the predictors by checking for the variance inflation factor, Variables exhibiting a VIF of 5 or higher were excluded from the analysis (Figure S1). This threshold helps to mitigate the impact of multicollinearity on the stability and interpretability of the regression coefficients. Multivariable linear regression models were used to estimate the associations between inflammatory indexs, respectively. This study set five different models (Model 1: unadjusted;

Model 2: age, sex, BMI, current smoking and current drinking, SBP , DBP;

Model 3: Model 2 plus adjustment for plus AST, ALT, HDL-C, LDL-C, TC, TG, PTH, ALP, Cr, FPG, serum potassium, serum calcium, serum phosphorus, 25-hydroxyvitamin D; Model 4: Model 3 plus adjustment for DM, CHD, and hypertension; Model 5: Model 4 plus adjustment for use of antihypertensive drug, antiplatelet medication, oral hypoglycemic drugs, insulin, statins). For each model, we calculated the odds ratio (OR) and the corresponding 95% confidence interval (CI) to quantify the strength and precision of the associations. The p-value for trend was determined by treating quartiles of the inflammatory index as a continuous variable within each model.

In addition, the restricted inverse square spline (RCS) (four nodes at the 5th, 35th, 65th, and 95th percentiles of the obesity metabolism index) was used to evaluate the dose-response relationship between obesity inflammatory index and the risks of osteoporosis. Finally, we employed receiver operating characteristic (ROC) and decision curve analysis (DCA) to determine their optimal diagnostic ability for osteoporosis. In addition, subgroup analyses were conducted by gender, age, BMI, hypertension current smoking and current drinking multiplicative interaction tests were applied. To ensure the robustness of our findings, we performed a series of sensitivity analyses to assess the stability of our results under different conditions. First, we conducted a sensitivity analysis to evaluate the impact of missing data by repeating the preliminary analysis after excluding subjects with incomplete covariate information at baseline. This approach allowed us to assess the potential bias that missing data might introduce into our estimates. Second, participants with BMI > 30 kg/m² were excluded because obesity itself may influence both inflammatory marker levels and bone metabolism, thereby acting as a potential confounder that could distort the true relationship between inflammation and osteoporosis risk. Third, individuals with DM were removed since DM is known to affect bone health and is associated with chronic inflammation, and thus could confound or modify the association of interest. Finally, we excluded participants of CHD, as CHD is frequently associated with systemic inflammation and altered bone metabolism, which might bias the association under investigation, and this result was still unaffected. All analyses were done using R (4.2.2).

All analyses were done using R (4.2.2). All P-values were two-sided, and P-values of <0.05 denoted statistical significance.

**2 Supplementary Tables**

**Table S1. List of medications included in the study**.

| Drug class | Drug name |
| --- | --- |
| Antihypertensive drug | Acetazolamide, amiloride, benzyl hydrochlorothiazide, bumetanide, furosemide, hydrochlorothiazide, indapamide, spironolactone, atenolol, bisoprolol, carvedilol, metoprolol, propranolol, amlodipine, diltiazem, felodipine, ercanidipine, nifedipine, verapamil, azilsartan, candestartan, captopril, enalapril, fosinopril, irbesartan, losartan, olmesartan, ramipril telmisartan, valsartan |
| Antiplatelet medication | Aspirin, clopidogrel, ticagrelor, prasugrel, dipyridamole, tirofiban, eptifibatide |
| Oral hypoglycemic drugs | Metformin, glipizide, gliclazide, glimepiride, repaglinide, nateglinide, acarbose, voglibose, pioglitazone, rosiglitazone  sitagliptin, vildagliptin, saxagliptin, empagliflozin, dapagliflozin, canagliflozin |
| Insulin | insulin lispro, insulin aspart, insulin glulisine, human insulin, protamine hagedorn, insulin glargine, insulin detemir, insulin degludec |
| Statins | Simvastatin, atorvastatin, pravastatin, rosuvastatin, lovastatin. pitavastatin, fluvastatin |

**Table S2. Sensitivity analyses of the association of six inflammatory indices with osteoporosis using a no-missing value data.**

| Exposure | Model 1 | Model 2 | Model 3 | Model 4 | Model 5 |
| --- | --- | --- | --- | --- | --- |
|  | OR (95% CI) | OR (95% CI) | OR (95% CI) | OR (95% CI) | OR (95% CI) |
| AISI (per SD increase) | 1.70 (1.54 - 1.88) | 1.73 (1.57 - 1.92) | 1.72 (1.55 - 1.91) | 1.73 (1.56 - 1.92) | 1.72 (1.55 - 1.91) |
| AISI quartiles |  |  |  |  |  |
| Q1 | Reference | Reference | Reference | Reference | Reference |
| Q2 | 1.48 (1.13 - 1.93) | 1.43 (1.07 - 1.91) | 1.43 (1.07 - 1.91) | 1.48 (1.13 - 1.94) | 1.47 (1.12 - 1.93) |
| Q3 | 2.37 (1.84 - 3.06) | 2.32 (1.76 - 3.06) | 2.29 (1.73 - 3.03) | 2.33 (1.80 - 3.03) | 2.29 (1.76 - 2.98) |
| Q4 | 4.46 (3.48 - 5.72) | 4.51 (3.44 - 5.91) | 4.43 (3.36 - 5.83) | 4.29 (3.31 - 5.55) | 4.25 (3.28 - 5.51) |
| P for trend | <0.001 | <0.001 | <0.001 | <0.001 | <0.001 |
| SIRI (per SD increase) | 1.68 (1.53 - 1.84) | 1.67 (1.52 - 1.83) | 1.66 (1.51 - 1.83) | 1.66 (1.51 - 1.83) | 1.66 (1.51 - 1.83) |
| SIRI quartiles |  |  |  |  |  |
| Q1 | Reference | Reference | Reference | Reference | Reference |
| Q2 | 1.50 (1.15 - 1.96) | 1.51 (1.16 - 1.98) | 1.49 (1.14 - 1.95) | 1.50 (1.14 - 1.96) | 1.49 (1.14 - 1.95) |
| Q3 | 2.34 (1.82 - 3.01) | 2.30 (1.78 - 2.97) | 2.27 (1.75 - 2.94) | 2.26 (1.74 - 2.93) | 2.25 (1.73 - 2.92) |
| Q4 | 4.11 (3.21 - 5.26) | 4.08 (3.17 - 5.24) | 4.03 (3.12 - 5.21) | 4.03 (3.12 - 5.20) | 4.00 (3.10 - 5.16) |
| P for trend | <0.001 | <0.001 | <0.001 | <0.001 | <0.001 |
| SII (per SD increase) | 1.51 (1.38 - 1.64) | 1.50 (1.37 - 1.65) | 1.50 (1.37 - 1.65) | 1.43 (1.31 - 1.56) | 1.45 (1.32 - 1.58) |
| SII quartiles |  |  |  |  |  |
| Q1 | Reference | Reference | Reference | Reference | Reference |
| Q2 | 1.71 (1.31 - 2.22) | 1.69 (1.27 - 2.25) | 1.65 (1.26 - 2.16) | 1.65 (1.26 - 2.16) | 1.61 (1.24 - 2.11) |
| Q3 | 2.57 (2.00 - 3.32) | 2.40 (1.82 - 3.16) | 2.42 (1.86 - 3.15) | 2.42 (1.85 - 3.15) | 2.30 (1.77 - 2.99) |
| Q4 | 3.38 (2.63 - 4.34) | 3.20 (2.42 - 4.21) | 3.10 (2.38 - 4.03) | 3.10 (2.38 - 4.03) | 2.98 (2.30 - 3.87) |
| P for trend | <0.001 | <0.001 | <0.001 | <0.001 | <0.001 |
| NLR (per SD increase) | 1.41 (1.32 - 1.52) | 1.42 (1.31 - 1.53) | 1.42 (1.31 - 1.54) | 1.38 (1.28 - 1.49) | 1.40 (1.29 - 1.51) |
| NLR quartiles |  |  |  |  |  |
| Q1 | Reference | Reference | Reference | Reference | Reference |
| Q2 | 1.49 (1.15 - 1.92) | 1.39 (1.05 - 1.84) | 1.50 (1.15 - 1.95) | 1.50 (1.15 - 1.95) | 1.46 (1.13 - 1.90) |
| Q3 | 2.22 (1.73 - 2.84) | 2.09 (1.60 - 2.73) | 2.24 (1.73 - 2.89) | 2.24 (1.73 - 2.89) | 2.15 (1.67 - 2.77) |
| Q4 | 3.14 (2.46 - 4.01) | 3.01 (2.30 - 3.93) | 2.95 (2.28 - 3.81) | 2.95 (2.29 - 3.82) | 2.83 (2.20 - 3.65) |
| P for trend | <0.001 | <0.001 | <0.001 | <0.001 | <0.001 |
| MLR (per SD increase) | 1.36 (1.28 - 1.44) | 1.38 (1.30 - 1.47) | 1.38 (1.29 - 1.47) | 1.37 (1.29 - 1.46) | 1.37 (1.29 - 1.46) |
| MLR quartiles |  |  |  |  |  |
| Q1 | Reference | Reference | Reference | Reference | Reference |
| Q2 | 1.47 (1.14 - 1.89) | 1.44 (1.12 - 1.86) | 1.43 (1.10 - 1.84) | 1.42 (1.10 - 1.84) | 1.43 (1.11 - 1.85) |
| Q3 | 2.01 (1.57 - 2.56) | 1.95 (1.52 - 2.50) | 1.92 (1.50 - 2.47) | 1.92 (1.49 - 2.46) | 1.91 (1.49 - 2.45) |
| Q4 | 2.96 (2.33 - 3.76) | 2.90 (2.28 - 3.71) | 2.81 (2.20 - 3.60) | 2.80 (2.19 - 3.59) | 2.80 (2.19 - 3.59) |
| P for trend | <0.001 | <0.001 | <0.001 | <0.001 | <0.001 |
| PLR (per SD increase) | 1.23 (1.17 - 1.29) | 1.19 (1.13 - 1.25) | 1.19 (1.13 - 1.25) | 1.19 (1.13 - 1.25) | 1.19 (1.13 - 1.25) |
| PLR quartiles |  |  |  |  |  |
| Q1 | Reference | Reference | Reference | Reference | Reference |
| Q2 | 1.53 (1.20 - 1.96) | 1.44 (1.12 - 1.84) | 1.43 (1.11 - 1.84) | 1.42 (1.11 - 1.83) | 1.42 (1.11 - 1.83) |
| Q3 | 1.90 (1.49 - 2.41) | 1.74 (1.35 - 2.23) | 1.72 (1.34 - 2.21) | 1.72 (1.34 - 2.21) | 1.73 (1.35 - 2.22) |
| Q4 | 2.32 (1.83 - 2.95) | 1.95 (1.51 - 2.52) | 1.95 (1.50 - 2.53) | 1.94 (1.50 - 2.52) | 1.95 (1.50 - 2.53) |
| P for trend | <0.001 | <0.001 | <0.001 | <0.001 | <0.001 |

Model 1: unadjusted;

Model 2: age, sex, BMI, current smoking and current drinking, SBP , DBP;

Model 3: Model 2 plus adjustment for plus AST, ALT, HDL-C, LDL-C, TC, TG, PTH, ALP, Cr, FPG, serum potassium, serum calcium, serum phosphorus, 25-hydroxyvitamin D;

Model 4: Model 3 plus adjustment for DM, CHD, and hypertension;

Model 5: Model 4 plus adjustment for use of antihypertensive drug, antiplatelet medication, oral hypoglycemic drugs, insulin, statins.

Abbreviations: SD, standard deviation; OR, odds ratio; CI, confidence interval. Other abbreviations are as defined in Table 1.

**Table S3. Sensitivity analysis of the association between six inflammatory indices and osteoporosis risk excluding participants after excluding patients with BMI>30 kg/m2.**

| Exposure | Model 1 | Model 2 | Model 3 | Model 4 | Model 5 |
| --- | --- | --- | --- | --- | --- |
|  | OR (95% CI) | OR (95% CI) | OR (95% CI) | OR (95% CI) | OR (95% CI) |
| AISI (per SD increase) | 1.80 (1.63 - 1.98) | 1.74 (1.58 - 1.92) | 1.68 (1.52 - 1.85) | 1.67 (1.52 - 1.85) | 1.67 (1.52 - 1.85) |
| AISI quartiles |  |  |  |  |  |
| Q1 | Reference | Reference | Reference | Reference | Reference |
| Q2 | 1.78 (1.35 - 2.34) | 1.79 (1.35 - 2.38) | 1.75 (1.32 - 2.33) | 1.73 (1.30 - 2.30) | 1.72 (1.29 - 2.29) |
| Q3 | 3.07 (2.36 - 3.98) | 3.03 (2.31 - 3.96) | 2.86 (2.17 - 3.75) | 2.85 (2.17 - 3.74) | 2.83 (2.15 - 3.72) |
| Q4 | 5.13 (3.97 - 6.64) | 4.97 (3.81 - 6.48) | 4.54 (3.46 - 5.96) | 4.49 (3.42 - 5.89) | 4.53 (3.45 - 5.94) |
| P for trend | <0.001 | <0.001 | <0.001 | <0.001 | <0.001 |
| SIRI (per SD increase) | 1.62 (1.50 - 1.75) | 1.64 (1.51 - 1.77) | 1.61 (1.49 - 1.75) | 1.61 (1.49 - 1.75) | 1.61 (1.49 - 1.75) |
| SIRI quartiles |  |  |  |  |  |
| Q1 | Reference | Reference | Reference | Reference | Reference |
| Q2 | 1.48 (1.15 - 1.91) | 1.46 (1.13 - 1.89) | 1.46 (1.11 - 1.92) | 1.45 (1.12 - 1.87) | 1.44 (1.12 - 1.87) |
| Q3 | 2.38 (1.87 - 3.03) | 2.44 (1.90 - 3.12) | 2.18 (1.67 - 2.85) | 2.23 (1.74 - 2.86) | 2.23 (1.73 - 2.86) |
| Q4 | 4.03 (3.19 - 5.10) | 4.17 (3.28 - 5.31) | 3.85 (2.98 - 4.99) | 4.03 (3.16 - 5.14) | 4.03 (3.16 - 5.14) |
| P for trend | <0.001 | <0.001 | <0.001 | <0.001 | <0.001 |
| SII (per SD increase) | 1.54 (1.42 - 1.67) | 1.53 (1.40 - 1.68) | 1.52 (1.39 - 1.67) | 1.52 (1.39 - 1.67) | 1.52 (1.39 - 1.67) |
| SII quartiles |  |  |  |  |  |
| Q1 | Reference | Reference | Reference | Reference | Reference |
| Q2 | 1.72 (1.33 - 2.23) | 1.60 (1.23 - 2.08) | 1.76 (1.33 - 2.33) | 1.61 (1.24 - 2.09) | 1.61 (1.24 - 2.09) |
| Q3 | 2.42 (1.89 - 3.10) | 2.25 (1.74 - 2.90) | 2.21 (1.68 - 2.90) | 2.14 (1.66 - 2.76) | 2.14 (1.66 - 2.77) |
| Q4 | 3.62 (2.84 - 4.60) | 3.19 (2.49 - 4.10) | 3.36 (2.57 - 4.38) | 3.12 (2.43 - 4.01) | 3.12 (2.43 - 4.02) |
| P for trend | <0.001 | <0.001 | <0.001 | <0.001 | <0.001 |
| NLR (per SD increase) | 1.43 (1.34 - 1.53) | 1.43 (1.33 - 1.53) | 1.43 (1.32 - 1.54) | 1.42 (1.32 - 1.53) | 1.42 (1.32 - 1.53) |
| NLR quartiles |  |  |  |  |  |
| Q1 | Reference | Reference | Reference | Reference | Reference |
| Q2 | 1.52 (1.19 - 1.94) | 1.54 (1.20 - 1.98) | 1.51 (1.17 - 1.94) | 1.50 (1.17 - 1.93) | 1.49 (1.16 - 1.92) |
| Q3 | 2.10 (1.65 - 2.66) | 2.08 (1.63 - 2.65) | 2.02 (1.59 - 2.58) | 2.02 (1.58 - 2.58) | 2.02 (1.59 - 2.58) |
| Q4 | 3.25 (2.58 - 4.10) | 3.21 (2.54 - 4.06) | 3.10 (2.44 - 3.93) | 3.09 (2.44 - 3.92) | 3.08 (2.43 - 3.91) |
| P for trend | <0.001 | <0.001 | <0.001 | <0.001 | <0.001 |
| MLR (per SD increase) | 1.41 (1.34 - 1.49) | 1.41 (1.33 - 1.49) | 1.41 (1.33 - 1.49) | 1.41 (1.33 - 1.49) | 1.40 (1.33 - 1.49) |
| MLR quartiles |  |  |  |  |  |
| Q1 | Reference | Reference | Reference | Reference | Reference |
| Q2 | 1.61 (1.26 - 2.06) | 1.54 (1.20 - 1.98) | 1.53 (1.17 - 2.01) | 1.56 (1.21 - 2.01) | 1.56 (1.21 - 2.01) |
| Q3 | 1.91 (1.50 - 2.43) | 1.87 (1.46 - 2.39) | 1.84 (1.41 - 2.40) | 1.82 (1.42 - 2.33) | 1.81 (1.41 - 2.32) |
| Q4 | 3.31 (2.62 - 4.17) | 3.04 (2.39 - 3.86) | 3.10 (2.40 - 4.00) | 2.90 (2.28 - 3.69) | 2.90 (2.28 - 3.69) |
| P for trend | <0.001 | <0.001 | <0.001 | <0.001 | <0.001 |
| PLR (per SD increase) | 1.24 (1.19 - 1.29) | 1.21 (1.15 - 1.27) | 1.21 (1.15 - 1.27) | 1.20 (1.15 - 1.26) | 1.20 (1.15 - 1.26) |
| PLR quartiles |  |  |  |  |  |
| Q1 | Reference | Reference | Reference | Reference | Reference |
| Q2 | 1.53 (1.20 - 1.95) | 1.44 (1.12 - 1.83) | 1.42 (1.10 - 1.82) | 1.41 (1.10 - 1.81) | 1.41 (1.10 - 1.81) |
| Q3 | 2.04 (1.61 - 2.58) | 1.85 (1.46 - 2.35) | 1.87 (1.47 - 2.39) | 1.87 (1.46 - 2.39) | 1.85 (1.45 - 2.36) |
| Q4 | 2.56 (2.04 - 3.22) | 2.13 (1.67 - 2.72) | 2.11 (1.64 - 2.72) | 2.12 (1.65 - 2.72) | 2.11 (1.64 - 2.71) |
| P for trend | <0.001 | <0.001 | <0.001 | <0.001 | <0.001 |

Model 1: unadjusted;

Model 2: age, sex, BMI, current smoking and current drinking, SBP , DBP;

Model 3: Model 2 plus adjustment for plus AST, ALT, HDL-C, LDL-C, TC, TG, PTH, ALP, Cr, FPG, serum potassium, serum calcium, serum phosphorus, 25-hydroxyvitamin D;

Model 4: Model 3 plus adjustment for DM, CHD, and hypertension;

Model 5: Model 4 plus adjustment for use of antihypertensive drug, antiplatelet medication, oral hypoglycemic drugs, insulin, statins.

Abbreviations: SD, standard deviation; OR, odds ratio; CI, confidence interval. Other abbreviations are as defined in Table 1.

**Table S4. Sensitivity analysis of the association between six inflammatory indices and osteoporosis risk excluding participants after excluding patients with DM.**

| Exposure | Model 1 | Model 2 | Model 3 | Model 4 | Model 5 |
| --- | --- | --- | --- | --- | --- |
|  | OR (95% CI) | OR (95% CI) | OR (95% CI) | OR (95% CI) | OR (95% CI) |
| AISI (per SD increase) | 1.81 (1.64 - 2.01) | 1.77 (1.59 - 1.96) | 1.77 (1.59 - 1.96) | 1.74 (1.56 - 1.93) | 1.73 (1.56 - 1.93) |
| AISI quartiles |  |  |  |  |  |
| Q1 | Reference | Reference | Reference | Reference | Reference |
| Q2 | 1.33 (1.02 - 1.73) | 1.34 (1.03 - 1.76) | 1.36 (1.04 - 1.78) | 1.36 (1.03 - 1.78) | 1.36 (1.04 - 1.79) |
| Q3 | 2.31 (1.80 - 2.96) | 2.35 (1.82 - 3.02) | 2.32 (1.80 - 3.00) | 2.27 (1.76 - 2.94) | 2.26 (1.75 - 2.92) |
| Q4 | 4.55 (3.57 - 5.79) | 4.43 (3.45 - 5.68) | 4.45 (3.46 - 5.71) | 4.29 (3.33 - 5.54) | 4.30 (3.34 - 5.56) |
| P for trend | <0.001 | <0.001 | <0.001 | <0.001 | <0.001 |
| SIRI (per SD increase) | 1.64 (1.52 - 1.78) | 1.68 (1.55 - 1.82) | 1.69 (1.55 - 1.83) | 1.67 (1.53 - 1.81) | 1.66 (1.53 - 1.81) |
| SIRI quartiles |  |  |  |  |  |
| Q1 | Reference | Reference | Reference | Reference | Reference |
| Q2 | 1.42 (1.09 - 1.84) | 1.42 (1.09 - 1.84) | 1.42 (1.09 - 1.85) | 1.42 (1.09 - 1.85) | 1.38 (1.05 - 1.83) |
| Q3 | 2.44 (1.90 - 3.13) | 2.43 (1.90 - 3.12) | 2.42 (1.87 - 3.12) | 2.41 (1.86 - 3.11) | 2.38 (1.81 - 3.11) |
| Q4 | 4.38 (3.44 - 5.58) | 4.36 (3.42 - 5.57) | 4.33 (3.37 - 5.56) | 4.30 (3.35 - 5.53) | 4.24 (3.25 - 5.51) |
| P for trend | <0.001 | <0.001 | <0.001 | <0.001 | <0.001 |
| SII (per SD increase) | 1.58 (1.46 - 1.72) | 1.50 (1.38 - 1.64) | 1.51 (1.38 - 1.65) | 1.50 (1.37 - 1.64) | 1.50 (1.37 - 1.64) |
| SII quartiles |  |  |  |  |  |
| Q1 | Reference | Reference | Reference | Reference | Reference |
| Q2 | 1.85 (1.42 - 2.41) | 1.86 (1.42 - 2.42) | 1.91 (1.45 - 2.50) | 1.91 (1.45 - 2.50) | 1.87 (1.41 - 2.49) |
| Q3 | 2.53 (1.96 - 3.27) | 2.55 (1.97 - 3.29) | 2.66 (2.05 - 3.45) | 2.65 (2.04 - 3.45) | 2.57 (1.95 - 3.39) |
| Q4 | 3.89 (3.03 - 5.00) | 3.92 (3.05 - 5.04) | 4.31 (3.32 - 5.59) | 4.28 (3.30 - 5.56) | 4.15 (3.16 - 5.46) |
| P for trend | <0.001 | <0.001 | <0.001 | <0.001 | <0.001 |
| NLR (per SD increase) | 1.46 (1.36 - 1.57) | 1.43 (1.33 - 1.54) | 1.43 (1.33 - 1.54) | 1.42 (1.32 - 1.54) | 1.43 (1.32 - 1.54) |
| NLR quartiles |  |  |  |  |  |
| Q1 | Reference | Reference | Reference | Reference | Reference |
| Q2 | 1.52 (1.18 - 1.96) | 1.52 (1.17 - 1.96) | 1.51 (1.16 - 1.96) | 1.51 (1.16 - 1.96) | 1.42 (1.08 - 1.87) |
| Q3 | 2.12 (1.65 - 2.71) | 2.11 (1.65 - 2.71) | 2.15 (1.67 - 2.76) | 2.14 (1.67 - 2.76) | 2.07 (1.59 - 2.69) |
| Q4 | 3.64 (2.86 - 4.63) | 3.62 (2.84 - 4.60) | 3.80 (2.96 - 4.87) | 3.78 (2.94 - 4.85) | 3.64 (2.81 - 4.73) |
| P for trend | <0.001 | <0.001 | <0.001 | <0.001 | <0.001 |
| MLR (per SD increase) | 1.40 (1.32 - 1.48) | 1.41 (1.33 - 1.50) | 1.42 (1.34 - 1.51) | 1.41 (1.33 - 1.50) | 1.41 (1.32 - 1.50) |
| MLR quartiles |  |  |  |  |  |
| Q1 | Reference | Reference | Reference | Reference | Reference |
| Q2 | 1.47 (1.14 - 1.90) | 1.47 (1.14 - 1.90) | 1.47 (1.14 - 1.91) | 1.47 (1.13 - 1.91) | 1.47 (1.14 - 1.91) |
| Q3 | 2.10 (1.64 - 2.68) | 2.11 (1.65 - 2.69) | 2.02 (1.58 - 2.59) | 2.03 (1.58 - 2.60) | 2.04 (1.59 - 2.62) |
| Q4 | 3.19 (2.51 - 4.05) | 3.19 (2.51 - 4.05) | 3.08 (2.41 - 3.93) | 3.07 (2.40 - 3.92) | 3.09 (2.42 - 3.95) |
| P for trend | <0.001 | <0.001 | <0.001 | <0.001 | <0.001 |
| PLR (per SD increase) | 1.35 (1.26 - 1.44) | 1.27 (1.18 - 1.37) | 1.28 (1.19 - 1.38) | 1.28 (1.19 - 1.38) | 1.28 (1.19 - 1.38) |
| PLR quartiles |  |  |  |  |  |
| Q1 | Reference | Reference | Reference | Reference | Reference |
| Q2 | 1.63 (1.26 - 2.11) | 1.63 (1.26 - 2.11) | 1.63 (1.26 - 2.10) | 1.45 (1.10 - 1.91) | 1.60 (1.24 - 2.05) |
| Q3 | 2.31 (1.79 - 2.97) | 2.30 (1.79 - 2.96) | 2.30 (1.78 - 2.95) | 1.88 (1.44 - 2.46) | 2.14 (1.68 - 2.73) |
| Q4 | 3.24 (2.50 - 4.18) | 3.23 (2.50 - 4.17) | 3.23 (2.50 - 4.17) | 2.69 (2.06 - 3.52) | 2.59 (2.04 - 3.29) |
| P for trend | <0.001 | <0.001 | <0.001 | <0.001 | <0.001 |

Model 1: unadjusted;

Model 2: age, sex, BMI, current smoking and current drinking, SBP , DBP;

Model 3: Model 2 plus adjustment for plus AST, ALT, HDL-C, LDL-C, TC, TG, PTH, ALP, Cr, FPG, serum potassium, serum calcium, serum phosphorus, 25-hydroxyvitamin D;

Model 4: Model 3 plus adjustment for CHD, and hypertension;

Model 5: Model 4 plus adjustment for use of antihypertensive drug, antiplatelet medication, statins.

Abbreviations: SD, standard deviation; OR, odds ratio; CI, confidence interval. Other abbreviations are as defined in Table 1.

**Table S5. Sensitivity analysis of the association between six inflammatory indices and osteoporosis risk excluding participants after excluding patients with CHD.**

| Exposure | Model 1 | Model 2 | Model 3 | Model 4 | Model 5 |
| --- | --- | --- | --- | --- | --- |
|  | OR (95% CI) | OR (95% CI) | OR (95% CI) | OR (95% CI) | OR (95% CI) |
| AISI (per SD increase) | 1.75 (1.59 - 1.94) | 1.70 (1.54 - 1.88) | 1.66 (1.50 - 1.83) | 1.66 (1.50 - 1.84) | 1.66 (1.50 - 1.84) |
| AISI quartiles |  |  |  |  |  |
| Q1 | Reference | Reference | Reference | Reference | Reference |
| Q2 | 1.76 (1.35 - 2.29) | 1.78 (1.35 - 2.34) | 1.78 (1.35 - 2.34) | 1.78 (1.35 - 2.33) | 1.76 (1.34 - 2.31) |
| Q3 | 3.17 (2.47 - 4.08) | 3.07 (2.36 - 3.98) | 3.07 (2.36 - 3.98) | 3.06 (2.36 - 3.97) | 3.05 (2.35 - 3.96) |
| Q4 | 5.16 (4.03 - 6.60) | 4.48 (3.45 - 5.80) | 4.48 (3.45 - 5.80) | 4.47 (3.45 - 5.80) | 4.50 (3.47 - 5.84) |
| P for trend | <0.001 | <0.001 | <0.001 | <0.001 | <0.001 |
| SIRI (per SD increase) | 1.69 (1.55 - 1.84) | 1.67 (1.52 - 1.84) | 1.65 (1.50 - 1.82) | 1.65 (1.50 - 1.82) | 1.62 (1.47 - 1.78) |
| SIRI quartiles |  |  |  |  |  |
| Q1 | Reference | Reference | Reference | Reference | Reference |
| Q2 | 1.37 (1.07 - 1.76) | 1.39 (1.07 - 1.79) | 1.37 (1.06 - 1.77) | 1.37 (1.06 - 1.77) | 1.36 (1.05 - 1.77) |
| Q3 | 2.38 (1.87 - 3.02) | 2.52 (1.97 - 3.22) | 2.39 (1.86 - 3.06) | 2.39 (1.86 - 3.06) | 2.36 (1.84 - 3.03) |
| Q4 | 3.89 (3.08 - 4.92) | 4.09 (3.21 - 5.20) | 3.96 (3.10 - 5.06) | 3.97 (3.11 - 5.07) | 3.93 (3.07 - 5.02) |
| P for trend | <0.001 | <0.001 | <0.001 | <0.001 | <0.001 |
| SII (per SD increase) | 1.52 (1.40 - 1.65) | 1.50 (1.37 - 1.64) | 1.49 (1.36 - 1.63) | 1.49 (1.36 - 1.63) | 1.49 (1.36 - 1.63) |
| SII quartiles |  |  |  |  |  |
| Q1 | Reference | Reference | Reference | Reference | Reference |
| Q2 | 1.72 (1.33 - 2.21) | 1.65 (1.26 - 2.17) | 1.62 (1.24 - 2.13) | 1.62 (1.24 - 2.13) | 1.62 (1.23 - 2.13) |
| Q3 | 2.51 (1.97 - 3.19) | 2.16 (1.66 - 2.80) | 2.13 (1.64 - 2.78) | 2.13 (1.63 - 2.77) | 2.13 (1.64 - 2.78) |
| Q4 | 3.38 (2.67 - 4.29) | 3.05 (2.36 - 3.95) | 3.01 (2.32 - 3.90) | 3.01 (2.32 - 3.90) | 3.02 (2.33 - 3.92) |
| P for trend | <0.001 | <0.001 | <0.001 | <0.001 | <0.001 |
| NLR (per SD increase) | 1.43 (1.34 - 1.54) | 1.41 (1.31 - 1.52) | 1.42 (1.31 - 1.53) | 1.42 (1.31 - 1.53) | 1.42 (1.32 - 1.53) |
| NLR quartiles |  |  |  |  |  |
| Q1 | Reference | Reference | Reference | Reference | Reference |
| Q2 | 1.48 (1.16 - 1.89) | 1.41 (1.09 - 1.84) | 1.40 (1.08 - 1.83) | 1.41 (1.08 - 1.83) | 1.41 (1.09 - 1.82) |
| Q3 | 2.03 (1.60 - 2.57) | 1.93 (1.50 - 2.49) | 1.92 (1.49 - 2.48) | 1.92 (1.49 - 2.48) | 1.89 (1.47 - 2.42) |
| Q4 | 3.16 (2.51 - 3.98) | 3.10 (2.42 - 3.96) | 3.07 (2.39 - 3.94) | 3.07 (2.39 - 3.94) | 2.94 (2.30 - 3.76) |
| P for trend | <0.001 | <0.001 | <0.001 | <0.001 | <0.001 |
| MLR (per SD increase) | 1.38 (1.30 - 1.46) | 1.39 (1.31 - 1.47) | 1.38 (1.30 - 1.47) | 1.38 (1.30 - 1.47) | 1.38 (1.30 - 1.47) |
| MLR quartiles |  |  |  |  |  |
| Q1 | Reference | Reference | Reference | Reference | Reference |
| Q2 | 1.47 (1.15 - 1.87) | 1.42 (1.10 - 1.85) | 1.42 (1.09 - 1.85) | 1.42 (1.09 - 1.84) | 1.45 (1.13 - 1.86) |
| Q3 | 2.07 (1.64 - 2.62) | 1.98 (1.54 - 2.55) | 1.97 (1.53 - 2.54) | 1.97 (1.53 - 2.54) | 2.01 (1.58 - 2.55) |
| Q4 | 3.11 (2.47 - 3.91) | 3.11 (2.44 - 3.98) | 3.01 (2.35 - 3.85) | 3.00 (2.34 - 3.84) | 2.91 (2.30 - 3.69) |
| P for trend | <0.001 | <0.001 | <0.001 | <0.001 | <0.001 |
| PLR (per SD increase) | 1.22 (1.17 - 1.28) | 1.18 (1.12 - 1.23) | 1.17 (1.12 - 1.23) | 1.17 (1.12 - 1.23) | 1.17 (1.12 - 1.23) |
| PLR quartiles |  |  |  |  |  |
| Q1 | Reference | Reference | Reference | Reference | Reference |
| Q2 | 1.54 (1.22 - 1.95) | 1.42 (1.11 - 1.80) | 1.40 (1.10 - 1.78) | 1.41 (1.10 - 1.79) | 1.38 (1.08 - 1.76) |
| Q3 | 1.94 (1.55 - 2.45) | 1.73 (1.36 - 2.19) | 1.72 (1.35 - 2.18) | 1.71 (1.35 - 2.18) | 1.66 (1.31 - 2.11) |
| Q4 | 2.35 (1.88 - 2.95) | 1.91 (1.49 - 2.43) | 1.88 (1.46 - 2.41) | 1.85 (1.45 - 2.37) | 1.81 (1.41 - 2.32) |
| P for trend | <0.001 | <0.001 | <0.001 | <0.001 | <0.001 |

Model 1: unadjusted;

Model 2: age, sex, BMI, current smoking and current drinking, SBP , DBP;

Model 3: Model 2 plus adjustment for plus AST, ALT, HDL-C, LDL-C, TC, TG, PTH, ALP, Cr, FPG, serum potassium, serum calcium, serum phosphorus, 25-hydroxyvitamin D;

Model 4: Model 3 plus adjustment for DM and hypertension;

Model 5: Model 4 plus adjustment for use of antihypertensive drug, antiplatelet medication, oral hypoglycemic drugs, insulin, statins.

Abbreviations: SD, standard deviation; OR, odds ratio; CI, confidence interval. Other abbreviations are as defined in Table 1.

**Table S6. Association between six inflammatory indices and osteoporosis tratified by geographic region.**

| Exposure | N | OR (95%CI) | P | P for interaction |
| --- | --- | --- | --- | --- |
| AISI (per SD increase) |  |  |  | 0.055 |
| Xinjang | 1598 (44.08) | 1.46 (1.30 ~ 1.64) | <0.001 |  |
| Sichuan | 1532 (42.26) | 1.60 (1.41 ~ 1.82) | <0.001 |  |
| Huangshan | 495 (13.66) | 1.26 (1.03 ~ 1.54) | 0.028 |  |
| SIRI (per SD increase) |  |  |  | 0.085 |
| Xinjang | 1598 (44.08) | 1.54 (1.37 ~ 1.74) | <0.001 |  |
| Sichuan | 1532 (42.26) | 1.84 (1.62 ~ 2.10) | <0.001 |  |
| Huangshan | 495 (13.66) | 1.45 (1.12 ~ 1.88) | 0.005 |  |
| SII (per SD increase) |  |  |  | 0.219 |
| Xinjang | 1598 (44.08) | 1.55 (1.40 ~ 1.73) | <0.001 |  |
| Sichuan | 1532 (42.26) | 1.56 (1.40 ~ 1.73) | <0.001 |  |
| Huangshan | 495 (13.66) | 1.22 (0.93 ~ 1.60) | 0.154 |  |
| NLR (per SD increase) |  |  |  | 0.241 |
| Xinjang | 1598 (44.08) | 1.28 (1.15 ~ 1.42) | <0.001 |  |
| Sichuan | 1532 (42.26) | 1.42 (1.25 ~ 1.63) | <0.001 |  |
| Huangshan | 495 (13.66) | 1.18 (0.97 ~ 1.44) | 0.095 |  |
| MLR (per SD increase) |  |  |  | 0.075 |
| Xinjang | 1598 (44.08) | 1.23 (1.10 ~ 1.36) | <0.001 |  |
| Sichuan | 1532 (42.26) | 1.42 (1.27 ~ 1.59) | <0.001 |  |
| Huangshan | 495 (13.66) | 1.10 (0.85 ~ 1.43) | 0.477 |  |
| PLR (per SD increase) |  |  |  | 0.708 |
| Xinjang | 1598 (44.08) | 1.34 (1.21 ~ 1.49) | <0.001 |  |
| Sichuan | 1532 (42.26) | 1.44 (1.28 ~ 1.61) | <0.001 |  |
| Huangshan | 495 (13.66) | 1.38 (1.08 ~ 1.75) | 0.009 |  |

Adjust: age, sex, BMI, current smoking and current drinking, SBP , DBP, AST, ALT, HDL-C, LDL-C, TC, TG, PTH, ALP, Cr, FPG, serum potassium, serum calcium, serum phosphorus, 25-hydroxyvitamin D, DM and hypertension, antihypertensive drug, antiplatelet medication, oral hypoglycemic drugs, insulin, statins

Abbreviations: SD, standard deviation; OR, odds ratio; CI, confidence interval. Other abbreviations are as defined in Table 1.

**Table S7. Incremental AUC improvement after adding AISI to the base clinical model**

| Model | Predictors Included | AUC (95% CI) | P |
| --- | --- | --- | --- |
| Base Model | Age + Sex + BMI | 0.775 (0.758 - 0.792) | Reference |
| Full Model | Base Model + AISI | 0.804 (0.788 - 0.821) | <0.001 |
